# Impact of glucose and pyruvate on adenosine triphosphate production and sperm motility in goats

**DOI:** 10.5713/ab.23.0229

**Published:** 2023-10-31

**Authors:** Rangga Setiawan, Raden Febrianto Christi, Ken Ratu Gharizah Alhuur, Rini Widyastuti, Nurcholidah Solihati, Siti Darodjah Rasad, Kundrat Hidajat, Duy Ngoc Do

**Affiliations:** 1 Department of Animal Production, Faculty of Animal Husbandry, Universitas Padjadjaran, Bandung-Sumedang KM 21, West Java 45363, Indonesia; 2 Department of Animal Science and Aquaculture, Dalhousie University, Truro, NS B2N 5E3, Canada

**Keywords:** Adenosine Triphosphate, Glycolysis, Goat, Oxidative Phosphorylation, Sperm

## Abstract

**Objective:**

This study evaluates goat sperm motility in response to metabolic substrates and various inhibitors, aiming to assess the relative contribution of glycolysis and mitochondrial oxidation for sperm movement and adenosine triphosphate (ATP) production.

**Methods:**

In the present study, two main metabolic substrates; 0 to 0.5 mM glucose and 0 to 30 mM pyruvate were used to evaluate their contribution to sperm movements of goats. Using a 3-chloro-1,2-propanediol (3-MCPD), a specific inhibitor for glycolysis, and carbonyl cyanide 3-chlorophenylhydrazone as an inhibitor for oxidative phosphorylation, cellular mechanisms into ATP-generating pathways in relation to sperm movements and ATP production were observed. Data were analysed using one-way analysis of variance for multiple comparisons.

**Results:**

Sperm motility analysis showed that either glucose or pyruvate supported sperm movement during 0 to 30 min incubation. However, the supporting effects were abolished by the addition of a glycolysis inhibitor or mitochondrial uncoupler, concomitant with a significant decrease in ATP production. Although oxidative phosphorylation produces larger ATP concentrations than those from glycolysis, sperm progressivity in relation to these two metabolic pathways is comparable.

**Conclusion:**

Based on the present study, we suggest that goat sperm use glucose and pyruvate to generate cellular energy through glycolysis and mitochondrial respiration pathways to maintain sperm movement.

## INTRODUCTION

Cellular energy is an essential factor that supports the functionality of spermatozoa. The sustenance of sperm motility during their traversal within the female reproductive tract hinges on consistent and enduring energy. This delineates a probable rationale for the augmented requirement of adenosine triphosphate (ATP) by spermatozoa, surpassing other cellular entities. ATP, as the quintessential embodiment of cellular energy, assumes paramount importance. Sperm motility is highly dependent on the availability of ATP compared to other sperm functions such as capacitation, acrosome reaction, or sperm penetrability, accounting since it accounts for approximately 70% of ATP consumption [1].

Sperm produce ATP through glycolysis and oxidative phosphorylation localized to different regions of the cells. The localization within the principal piece underscores the significance of glycolysis in providing comprehensive support for tyrosine phosphorylation and hyperactivated motility. Several glycolytic enzymes have been identified to be situated within the fibrous sheath of the principal piece [2]. Oxidative phosphorylation occurs in the mitochondria, which are tightly packed in the midpiece producing abundant ATP. Even though oxidative phosphorylation produces more ATP than glycolysis, there is a wide range of variation among species regarding the relative dependency of ATP utilization. For example, the flagellar movement of murine and human sperm is supported by ATP generated from glycolysis, but this movement in boar and horses mainly uses oxidative phosphorylation [3–5]. For goat sperm, the mechanism of ATP generation is still unknown.

Glucose is the primary substrate for glycolysis. It is converted into pyruvates and energy, including a total of 4 ATP, 2 nicotineamide adenine dinucleotide (NADH), and 2 pyruvates per glucose molecule [6]. The pyruvates were used as a source for oxidative phosphorylation producing 32 ATP molecules [7]. Some studies reported the effects of pyruvate and glucose utilization on sperm motility varying between species. Results in roosters indicated sperm motility requires ATP, resulting from glucose uptake and oxidative phosphorylation [8]. It is reported that glucose increases sperm velocity, increasing penetrability into eggs [9] but sperm motility and velocity depend on pyruvate in stallions and boar [10,11]. Inadequate lactate and pyruvate as metabolic substrates for Oxphos lead to mitochondrial dysfunction resulting in increased reactive oxygen species production and decreased motility [11]. However, pyruvate does not affect sperm penetration into eggs in rodents. Despite these findings, it has been unclear and debated in the field whether glycolysis or oxidative phosphorylation is the major contributor to ATP needed for sperm motility in goats. The present study evaluated the major contributor of ATP production in the relationship with sperm motility in goats.

## MATERIALS AND METHODS

### Reagents and animals

All chemicals, such as tris (hydroxymethyl), citric acid, D-glucose, natrium pyruvate, 3-chloro-1,2-propanediol (3-MCPD) as a glycolysis inhibitor, carbonyl cyanide 3-chlorophenylhydrazone (CCCP) as mitochondrial uncoupler were purchased from Sigma-Aldrich (St. Louis, CA, USA). Meanwhile, the ATP bioluminescence assay kit II was purchased from Roche (Mannheim, Germany).

Semen was collected from 3 fertile male Ettawa Crossbred goats using an artificial vagina. Furthermore, the semen from each buck was washed twice by centrifugation at 250 *g* for 5 min at RT with tris-citrate buffer (pH 7.2) to exclude seminal plasma. All animal work was performed under the approval of the Research Ethics Committee of the Universitas Padjadjaran (approval no. 39/UN6.KEP/EC/2023).

### Sperm incubation and motility

A total of 10^7^ sperm samples were incubated in tris-citrate buffer (332 mM tris, 83 mM citrate, pH 7.2) at 37°C with 0 to 2 mM glucose, 0 to 30 mM pyruvate, 0 to 2 mM 3-MCPD, and 0 to 80 μM CCCP for 0, 15, and 30 min. In addition, 3-MCPD (a glycolysis inhibitor) and CCCP (a proton ionophore) were used to inhibit glycolysis and mitochondrial oxidative phosphorylation, respectively. Sperm motility was recorded using digital video microscopy for 5 s and was categorized as progressive, non-progressive, and non-motile [12]. In brief, sperm that have forward progression (forward and slow) and sluggish motility were categorized as progressive motile sperm; non-progressive motile sperm when sperm that vibrates in place; non-motile for sperm with no movement; and total motile sperm was the sum of progressive and non-progressive motile sperm. Sperm motility profiles were then observed for spermatozoa incubated in combination with 0.5 mM glucose, 5 mM pyruvate, 0.5 mM 3-MCPD, and 40 μM CCCP at 37°C for 30 min to determine the relative dependency of sperm motility on ATP providers.

### ATP quantification

ATP concentration was quantified using the ATP bioluminescence assay kit II (Roche, Germany). Sperm (1×10^7^) were incubated with 0- or 0.5-mM glucose, 5 mM pyruvate, 0.5 mM 3-MCPD, and 40 μM CCCP in Tris-citrate solution at 37°C for 30 min. After washing with Tris-citrate solution, sperm were solubilized with lysis reagent and incubated at 25°C for 5 min. The supernatant was transferred to a fresh tube and mixed with luciferase reagent after centrifugation at 10,000 g for 1 min. The bioluminescence signal was measured using a multimode Tecan infinite M200PRO plate reader.

### Statistical analysis

Multiple comparisons were performed using one-way analysis of variance, followed by Tukey’s honestly significant difference. Results were expressed as mean±standard error of the mean and the differences were considered significant when there was a p-value of <0.05.

## RESULTS

The effect of glucose and incubation period was investigated by incubating sperm in 0, 0.5, 1, or 2 mM of glucose for 0, 15, and 30 min at 37°C. The results showed that there were no differences in total motile (77.2% to 84.5%), motile progressive (66.9% to 76.7%), and non-motile (15.5% to 23.6%) when sperm were incubated in different levels of glucose for 0 to 15 min. However, non-progressive motility in the group of sperm without glucose supplementation (4.5% compared with 17.6%) showed a difference. Total motile (79.0% compared with 45.4%) and motile progressive (63.8% compared with 31.1%) were significantly greater in sperm incubated with 0.5 mM glucose than no glucose for 30 min (p<0.05; Table 1). There were no significant differences (p>0.05) in non-progressive motile between glucose and no glucose supplementation groups in 30 min incubation (14.4% to 16.7%). Sperm without glucose supplementation had the highest non-motile sperm (54.6% compared with 21% to 26%). The addition of 1 mM and 2 mM glucose did not result in a further increase in the value of sperm motility variables. Therefore, glucose has roles in sperm motility regulation as a glycolytic substrate, as shown in Table 1.

To examine the effects of pyruvate as an oxidative phosphorylation substrate, sperm were subjected to motility analysis by visual examination immediately (0 min) or after incubation for 15 and 30 min with 0-, 5-, 10-, or 30-mM pyruvate supplementation. The sperm motility characteristics at 0 min were as follows: total motile, 81.9%; motile progressive, 75.2%; non-progressive motile, 6.6%; and non-motile, 18.1%. Total motile (15 min, 75.8%; 30 min, 49.2%), motile progressive (15 min, 71.0%; 30 min, 36.9%), non-progressive motile (15 min, 4.8%; 30 min, 12.3%), and non-motile (15 min, 24.2%; 30 min, 50.8%) decreased in a time-dependent manner (p<0.05) when sperm were incubated without pyruvate supplementation. Meanwhile, no changes were observed for all sperm motility characteristics when sperm were incubated with or without pyruvate for 15 min. The addition of 5 to 30 mM pyruvate showed a greater value in total motile and motile progressive compared to the control with a prolonged incubation time of up to 30 min. There were no significant differences in non-progressive motile sperm incubated with or without pyruvate, but sperm without pyruvate had the highest value in non-motile. Interestingly, the addition of 5 mM pyruvate maintained total motile (30 min, 75% compared with 0 min, 81.9%), motile progressive (30 min, 65.7% compared with 0 min, 75.2%), non-progressive motile (30 min, 9.3% compared with 0 min, 6.6%) and non-motile (30 min, 25.0% compared with 0 min, 18.1%) (Table 2). These results suggested the involvement of pyruvate in goat sperm movement.

**Figure 1:**
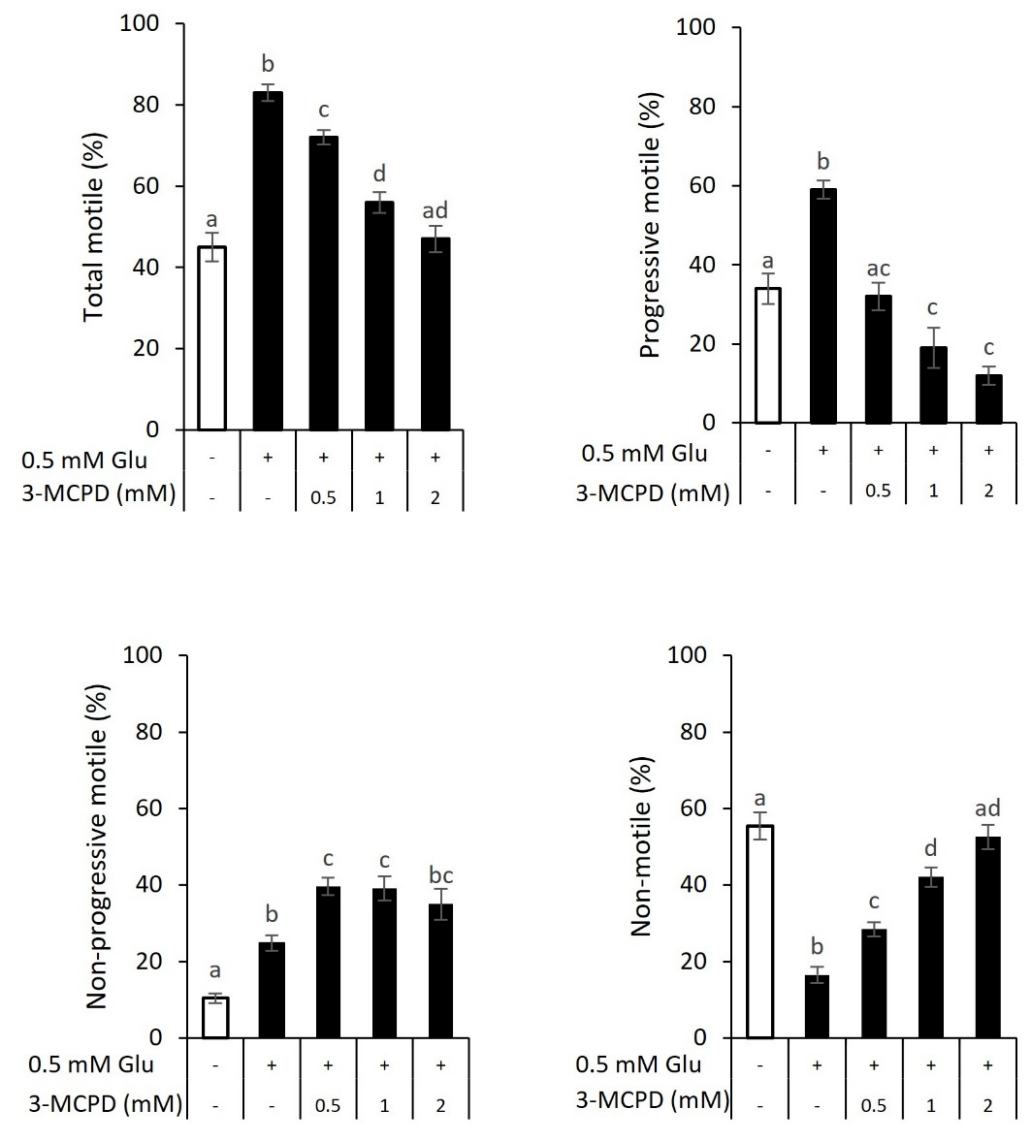
Motility characteristics of sperm incubated for 30 min in the presence of 3-chloro-1,2-propanediol (3-MCPD) with or without 0.5 mM glucose supplementation. Spermatozoa were incubated with 0.5, 1, or 2 mM 3-MCPD with 0- or 0.5-mM glucose supplementation for 30 min. Total and progressive motile were increased by glucose supplementation, but decreased in the presence of 0.5, 1, or 2 mM 3-MCPD. Contrarily, non-motile sperm was low in the presence of glucose but increased by 3-MCPD. Data are expressed as the mean±standard error of the mean (n = 6). ^a–d^ Different letters above columns indicate significant differences (p<0.05).

To examine glucose-dependent motility through glycolysis, sperm were incubated with 0, 0.5, 1, or 2 mM 3-MCPD for 30 min in the presence of 0.5 mM, followed by visual examination for motility analysis. When sperm were incubated without 3-MCPD, values for total motile, progressive motile, and non-progressive motile (Figure 1; 83.5%, 58.5%, and 24.9%, respectively) were larger in sperm with glucose supplementation than in sperm without glucose (Figure 1; 44.6%, 34.2%, and 10.4%, respectively). Only non-motile sperm with glucose (16.5%) were less than those without glucose supplementation (55.4%). However, the presence of 3-MCPD decreased glucose-dependent total motile (0.5 mM, 71.6%; 1 mM, 57.9%; 2 mM, 47.4%; respectively) and progressive-motile sperm (0.5 mM, 32.0%; 1 mM, 18.8%; 2 mM, 12.3%; respectively). The addition of 3-MCPD increased non-progressive motile (0.5 mM, 39.6%; and 1 mM, 39.1%; except 2 mM, 35.0%; respectively) compared with sperm with glucose (24.9%). The addition of 3-MCPD also increased non-motile sperm (0.5 mM, 28.4%; 1 mM, 42.1%; 2 mM, 52.6%; respectively) compared with sperm incubated with glucose (16.5%).

**Figure 2:**
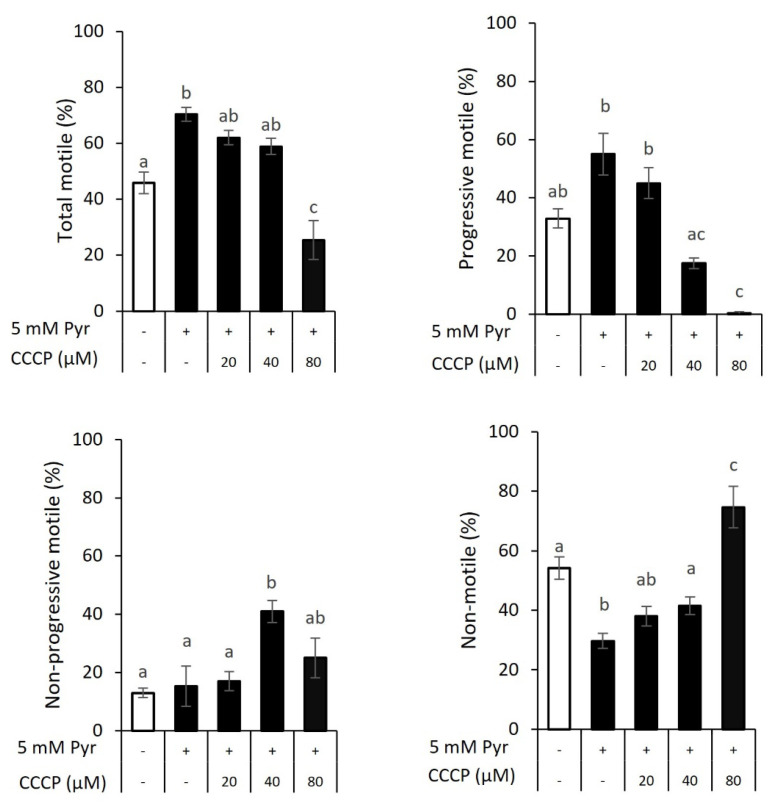
Motility characteristics of sperm incubated for 30 min in the presence of carbonyl cyanide 3-chlorophenylhydrazone (CCCP) with or without 5 mM pyruvate supplementation. Spermatozoa were incubated with 20, 40, or 80 μM CCCP in Tris-citrate with or without 5 mM pyruvate for 30 min. Pyruvate supplementation increased the total and progressive motile of sperm, but the presence of CCCP abolished the increasing effect of pyruvate at 80 μM for total motile and 40 to 80 μM for progressive motile of sperm. No increasing effect of pyruvate was found on non-progressive motile, but the presence of 40 μM CCCP increased the percentage of non-progressive motile. The lowest percentage of non-motile sperm was found in the presence of pyruvate, but it is significantly increased by 80 μM CCCP. Data are expressed as the mean±standard error of the mean (n = 6). ^a–c^ Different letters above columns indicate significant differences (p<0.05).

The importance of oxidative phosphorylation in goat sperm motility was examined by incubating sperm with 0, 20, 40, or 80 μM CCCP for 30 min in the presence of 5 mM pyruvate, followed by visual examination for motility analysis. The total and progressive motile values of sperm incubated with 5 mM pyruvate were larger (Figure 2; 70.3% and 55.0%, respectively) than those without pyruvate supplementation (45.8% and 32.9%, respectively). There were no significant differences in total motile when sperm incubated with 20 to 40 μM CCCP (62.0% to 58.5%) and progressive motile in 20 μM CCCP (45%) compared with pyruvate-added sperm. However, the addition of 40 μM CCCP decreased the progressive motile of sperm by 17.5%. The addition of 80 μM CCCP drastically decreased both total and progressive motile (25.4% and 0.4%, respectively). Furthermore, in response to 40 μM CCCP, non-progressive motile (41.0%) was higher than that of sperm incubated without pyruvate and CCCP (12.9%), with pyruvate (15.2%), or pyruvate and 20 μM CCCP supplementation (17.0%) but did not differ from 80 μM CCCP group (25.0%). Likewise, the presence of CCCP increased non-motile sperm in a dose-dependent manner, indicating that mitochondrial respiration took part in the energy supply for the motility of goat sperm.

**Figure 3:**
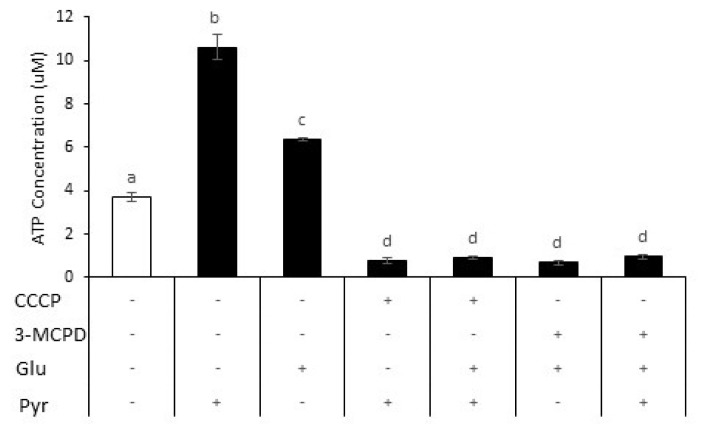
ATP Concentration in response to metabolic substrate supplementation and inhibitors. ATP concentrations were increased either by glucose or pyruvate supplementation but decreased when 3-chloro-1,2-propanediol (3-MCPD) or carbonyl cyanide 3-chlorophenylhydrazone (CCCP) were added. Data are expressed as mean±standard error of the mean (n = 4). ^a–d^ Different letters above columns indicate significant differences (p<0.05).

To examine the role of glycolysis and oxidative phosphorylation in goat sperm ATP production, cellular ATP concentration was quantified in sperm incubated with 3-MCPD, CCCP, with or without glucose and pyruvate supplementation. In the presence of 5 mM pyruvate, the ATP concentration of sperm increased (mean increase of 185%), while 0.5 mM glucose increased ATP content (mean increase 72%) compared to those in sperm without sugars supplementation (Figure 3). The addition of inhibitors, either 3-MCPD or CCCP decreased ATP content for all treatments (91% to 93% vs pyruvate addition; 85% to 89% vs glucose addition), suggesting that pyruvate and glucose play a role in ATP production. There were no significant differences in ATP content among inhibitor groups.

**Figure 4:**
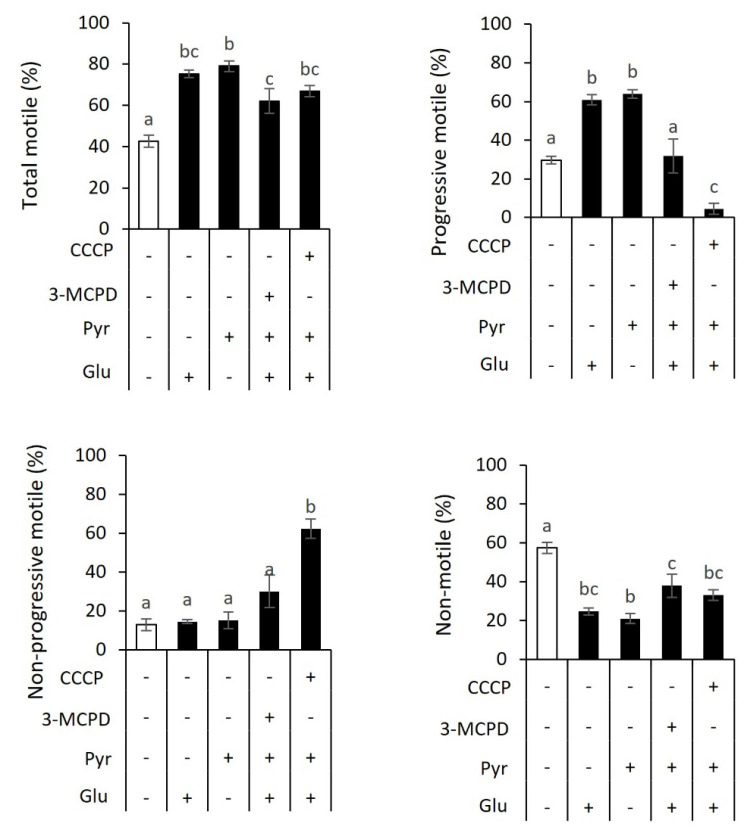
Changes in sperm motility profile in response to metabolic substrates and inhibitors. The sperm motility analysis revealed increased total and progressive motility in response to glucose or pyruvate supplementation. 3-Chloro-1,2-propanediol (3-MCPD) or carbonyl cyanide 3-chlorophenylhydrazone (CCCP) decreased total and progressive motile, even in the presence of both glucose and pyruvate. Non-progressive motile sperm tend to increase as a consequence of reduced progressive motile by inhibitors. Data are expressed as mean±standard error of the mean (n = 6). ^a–c^ Different letters above columns indicate significant differences (p<0.05).

The relative dependency of sperm motility on ATP providers was determined by incubating sperm with the combination of 0.5 mM glucose, 5 mM pyruvate, 0.5 mM 3-MCPD, and 40 μM CCCP supplementation (Figure 4). Consistent with the findings in Figures 1 and 2, incubation with either glucose or pyruvate improved total motile (75.3% or 79.0%, respectively) and progressive motile (60.9% or 63.9%, respectively) compared with those in control (total motile, 42.6%; progressive motile, 29.7%). There were no differences in non-progressive motile sperm (control, 12.9%; glucose, 14.5%; and pyruvate, 15.2%). However, non-motile sperm were lower in sperm with glucose (24.7%) and pyruvate (21.0%) than control (57.4%) (p<0.05).

Adding 3-MCPD in a buffer containing glucose and pyruvate resulted in a lower total motile (62.0%) than pyruvate-added sperm (79.0%). However, there was no difference with glucose-added sperm (75.3%), indicating total motile sperm might be compensated by oxidative phosphorylation. The treatment group also resulted in a lesser progressive motile sperm (31.9%) compared with glucose-added sperm (60.9%) and pyruvate-added sperm (63.9%). There were no differences in non-progressive motile in the treatment group of 3-MCPD (30.1%), glucose (14.5%), and pyruvate (15.2%), but 3-MCPD increased non-motile sperm (38%) compared with pyruvate group (21%). The presence of CCCP in a media containing glucose and pyruvate dramatically decreased progressive motile sperm (4.5%) compared with glucose-added sperm (60.9%) and pyruvate-added sperm (63.9%). It increased non-progressive motile sperm (63.9%) compared with those in glucose treatment group (14.5%) and pyruvate treatment group (15.2%). There was no difference in total motile between the CCCP (66.8%), glucose (75.3%), and pyruvate treatment groups (79.0%), as well as in non-motile sperm (33.2%, 24.7%, and 21.0%, respectively). These results suggested that glycolysis and oxidative phosphorylation were energy providers for sperm motility in goats.

## DISCUSSION

Sperm motility is highly dependent on the availability of cellular ATP. Despite the importance of metabolic substrates such as glucose and pyruvate for ATP production and sperm functions in goats, the regulatory mechanisms involved in energy metabolisms for sperm motility are poorly understood. We found that ATP supports sperm motility in goats through glycolysis and oxidative phosphorylation. The results of the present study provide new insights into ATP-generating pathways that support flagellar motility in goats.

Goat uterine fluid contains 0.5 mM of glucose [13], but the pyruvate concentration in the female reproductive tract is unknown. A previous study showed that 5 to 10 mM of pyruvate-maintained sperm progressivity for up to 3 days of storage in goats [14]. Considering glucose concentration in uterine fluid and the effect of pyruvate concentration on sperm motility, the present study evaluated the effect of 0 to 2 mM glucose and 0 to 30 mM pyruvate on values of sperm motility variables. The concentration of 0.5 mM glucose was sufficient to maintain total motile, progressive motile, and non-motile sperm for up to 30 min incubation. These results were consistent with previous studies where glucose was reported to support the flagellar motility of sperm in different species [8,15,16]. Previously, several glucose transporters were localized in the flagellar region in mammalian spermatozoa [17,18]. The present study also found that the supplementation of 5 mM pyruvate was sufficient to support total motile, progressive motile, and maintain low non-motile sperm. Pyruvate was also transported by monocarboxylate transporters across biological membranes and was metabolized through electron transfer in the respiratory chain to support motility [14,19]. Even though glucose and pyruvate were important metabolic substrates for flagellar movements of sperm in goats, the cellular mechanisms should be analyzed.

In mammalian spermatozoa, energy metabolisms supporting flagellar motility depend on glycolysis in the principal piece and oxidative phosphorylation in the mitochondria [4]. Since 3-MCPD is a potent inhibitor of glyceraldehyde 3-phosphate dehydrogenase and CCCP is an uncoupler of mitochondrial oxidative phosphorylation, the effects were evaluated at 0 to 2 mM and 0 to 80 μM in the presence of glucose and pyruvate. The glycolysis inhibitor at ≥0.5 mM dramatically reduced total motile and progressive motile. A marked reduction in progressive motile was observed in the presence of ≥40 μM CCCP. In line with a previous study regarding the inhibitory effect of glycolysis using iodoacetate and mitochondrial respiration using rotenone or alpha-cyano-4-hydroxycinnamate (4-CIN) on sperm motility in goats [14], our results indicate the functional importance of glycolysis and oxidative phosphorylation in the flagellar movements of goat sperm.

In the present study, total motile was decreased by 3-MCPD in the presence of glucose alone but slightly improved when pyruvate was also present. Similarly, the inhibition of total motile by CCCP with pyruvate alone was partially increased by the addition of glucose. The compensatory effect from either glucose or pyruvate addition was not observed in progressive motile. However, the suppression of mitochondrial respiration had greater efficacy in diminishing the population of progressively motile sperm, in comparison to the suppression of glycolysis. This observation implied the disparate roles of energy metabolic pathways in governing the flagellar motion of sperm. Several studies have documented functional differences in energy metabolic pathways on sperm motility in different species. For example, sperm motility in stallions depends heavily on oxidative phosphorylation [5], and glycolysis is the main ATP-generating pathway for sperm motility in mice [3].

Results from a previous study of mouse sperm indicated higher cytoplasmic ATP content associated with higher swimming velocities [20]. Even though pyruvate-added sperm produce more ATP concentration than glucose, sperm progressivity between the two metabolic substrates is comparable, which might suggest that ATP produced from glycolysis is efficient and effective in supporting the flagellar movement. Because flagellar movement is caused by the activity of dynein ATPase that is localized along the entire length of the flagellum and depends on the ATP supply, it is reasonable that glycolysis occurred in the principal piece of the flagellum predominantly provides ATP for dynein ATPase of the flagella. Oxidative phosphorylation produces a large amount of ATP in the mitochondria but does not sufficiently propagate in the principal piece to support flagellar movement [3]. Furthermore, low ATP content was detected in sperm incubated either with 3-MCPD or CCCP, resulting in low progressive motility, even though glucose or/and pyruvate were present, suggesting either glycolysis or oxidative phosphorylation can independently support sperm flagellar movement in goats. It was hypothesized that sperm could generate ATP from glycolysis when the substrates were present. Conversely, when there were oxidative phosphorylation substrates, sperm used those substrates through mitochondrial respiration in the midpiece to provide ATP for flagellar movement. The mechanisms of ATP generation by oxidative phosphorylation distributed to the entire flagellum should be investigated. In a preceding investigation concerning sea urchin sperm, the phosphocreatine shuttle assumed the responsibility of conveying ATP from the mitochondrion to the remote flagellum [21]. This finding propelled the suggestion of a prospective inquiry into the functional attributes of phosphocreatine shuttle within goat sperm.

## CONCLUSION

Our study demonstrates the importance of glucose and pyruvate in supporting sperm motility via glycolysis and oxidative phosphorylation. Although oxidative phosphorylation produces larger ATP than glycolysis, sperm motility in response to both ATP-generating pathways is comparable. Results from the present study provide a new insight into the functional nature of the utilization of extra-cellular metabolic substrates in goat sperm to produce cellular energy for sperm movement that is enhanced in the development of sperm preservation.

## Figures and Tables

**Table 1:** Changes in sperm movement characteristics following incubation for 0 to 30 min with different levels of glucose (mM) supplementation

Item (%)	0 min	15 min				30 min			
		
	Control	Control	0.5	1	2	Control	0.5	1	2
Total motile	81.4±1.3^a^	77.2±3.7^a^	84.5±1.2^a^	81.3±1.9^a^	76.4±4.5^a^	45.4±3.2^b^	79.0±2.3^a^	79.8±1.9^a^	73.5±1.6^a^
Motile progressive	68.3±6.3^ab^	72.6±3.4^ab^	66.9±6.0^ab^	76.7±0.8^a^	70.8±4.5^ab^	31.1±3.4^c^	63.8±3.2^ab^	63.1±4.6^ab^	59.3±1.8^b^
Non-progressive motile	13.1±5.3^ab^	4.5±1.1^b^	17.6±5.4^a^	4.6±1.5^b^	5.6±0.7^b^	14.4±3.0^ab^	15.2±2.2^a^	16.7±1.7^a^	14.2±2.5^ab^
Non-motile	18.6±1.2^a^	22.8±3.6^a^	15.5±1.2^a^	18.7±1.9^a^	23.6±4.5^a^	54.6±3.2^b^	21.0±2.3^a^	20.2±4.9^a^	26.5±1.5^a^

a–c Data are expressed as the mean±standard error of the mean (n = 6). Within rows, different letters indicate a significant difference (p<0.05).

**Table 2:** Changes in sperm movement characteristics following incubation for 0 to 30 min with different levels of pyruvate (mM) supplementation

Items (%)	0 min	15 min				30 min			
		
	Control	Control	5	10	30	Control	5	10	30
Total motile	81.9±0.8^a^	75.8±1.9^ab^	77.8±1.8^ab^	78.5±1.0^ab^	78.4±1.0^ab^	49.2±3.3^c^	75.0±1.4^a^	73.6±1.0^b^	71.3±1.2^b^
Motile progressive	75.2±2.9^a^	71.0±2.3^ab^	69.8±1.1^ab^	70.9±3.0^ab^	67.0±1.0^ab^	36.9±3.7^c^	65.7±3.3^ab^	62.2±4.3^b^	62.1±3.8^b^
Non-progressive motile	6.6±2.6^a^	4.8±1.4^a^	8.0±2.0^a^	7.5±2.2^a^	11.4±1.0^a^	12.3±3.7^a^	9.3±2.6^a^	11.4±5.1^a^	9.2±2.8^a^
Non-motile	18.1±0.8^a^	24.2±1.9^ab^	22.2±1.8^ab^	21.5±1.0^ab^	21.6±1.0^ab^	50.8±3.3^c^	25.0±1.4^a^	26.4±2.6^b^	28.7±1.2^b^

a–c Data are expressed as the mean±standard error of the mean (n = 6). Within rows, different letters indicate a significant difference (p<0.05).

## References

[1] Bohnensack R, Halangk W (1986). Control of respiration and of motility in ejaculated bull spermatozoa. Biochim Biophys Acta Bioenerg.

[2] Turner RM (2005). Moving to the beat: a review of mammalian sperm motility regulation. Reprod Fertil Dev.

[3] Mukai C, Okuno M (2004). Glycolysis plays a major role for adenosine triphosphate supplementation in mouse sperm flagellar movement. Biol Reprod.

[4] Ford WCL (2006). Glycolysis and sperm motility: does a spoonful of sugar help the flagellum go round?. Hum Reprod Update.

[5] Davila MP, Muñoz PM, Tapia JA (2015). Inhibition of mitochondrial complex I leads to decreased motility and membrane integrity related to increased hydrogen peroxide and reduced ATP production, while the inhibition of glycolysis has less impact on sperm motility. PLoS One.

[6] Chaudhry R, Varacallo M (2018). Biochemistry, glycolysis.

[7] Melkonian EA, Schury MP (2019). Biochemistry, anaerobic glycolysis.

[8] Setiawan R, Priyadarshana C, Tajima A, Travis AJ, Asano A (2020). Localisation and function of glucose transporter GLUT1 in chicken (Gallus gallus domesticus) spermatozoa: relationship between ATP production pathways and flagellar motility. Reprod Fertil Dev.

[9] Setiawan R, Priyadarshana C, Miyazaki H, Tajima A, Asano A (2021). Functional difference of ATP-generating pathways in rooster sperm (Gallus gallus domesticus). Anim Reprod Sci.

[10] Brooks DE, Mann T (1973). Pyruvate metabolism in boar spermatozoa. J Reprod Fertil.

[11] Darr CR, Varner DD, Teague S (2016). Lactate and pyruvate are major sources of energy for stallion sperm with dose effects on mitochondrial function, motility, and ROS production. Biol Reprod.

[12] Elia J, Imbrogno N, Delfino M, Mazzilli R, Rossi T, Mazzilli F (2010). The importance of the sperm motility classes-future directions. Open Androl J.

[13] Wittek T, Erices J, Elze K (1998). Histology of the endometrium, clinical–chemical parameters of the uterine fluid and blood plasma concentrations of progesterone, estradiol-17β and prolactin during hydrometra in goats. Small Rumin Res.

[14] Qiu JH, Li YW, Xie HL (2016). Effects of glucose metabolism pathways on sperm motility and oxidative status during long-term liquid storage of goat semen. Theriogenology.

[15] Mujica A, Moreno-Rodriguez R, Naciff J, Neri L, Tash JS (1991). Glucose regulation of guinea-pig sperm motility. Reproduction.

[16] Ponglowhapan S, Essén-Gustavsson B, Forsberg CL (2004). Influence of glucose and fructose in the extender during long-term storage of chilled canine semen. Theriogenology.

[17] Bucci D, Rodriguez-Gil JE, Vallorani C, Spinaci M, Galeati G, Tamanini C (2011). GLUTs and mammalian sperm metabolism. J Androl.

[18] Bucci D, Isani G, Spinaci M (2010). Comparative immunolocalization of GLUTs 1, 2, 3 and 5 in boar, stallion and dog spermatozoa. Reprod Domest Anim.

[19] Halestrap AP, Meredith D (2004). The SLC16 gene family - From monocarboxylate transporters (MCTs) to aromatic amino acid transporters and beyond. Pflugers Arch Eur J Physiol.

[20] Tourmente M, Villar-Moya P, Rial E, Roldan ERS (2015). Differences in ATP generation via glycolysis and oxidative phosphorylation and relationships with sperm motility in mouse species. J Biol Chem.

[21] Tombes RM, Shapiro BM (1985). Metabolite channeling: a phosphorylcreatine shuttle to mediate high energy phosphate transport between sperm mitochondrion and tail. Cell.

